# Effect of PTGES3 on the Prognosis and Immune Regulation in Lung Adenocarcinoma

**DOI:** 10.1155/2023/4522045

**Published:** 2023-06-28

**Authors:** Wenyan Jiang, Qiong Wei, Haiqin Xie, Dandan Wu, Haiyan He, Xuedong Lv

**Affiliations:** Department of Respiratory Medicine, The Second Affiliated Hospital of Nantong University, Nantong 226001, China

## Abstract

**Background:**

PTGES3 is upregulated in multiple cancer types and promotes tumorigenesis and progression. However, the clinical outcome and immune regulation of PTGES3 in lung adenocarcinoma (LUAD) are not fully understood. This study aimed to explore the expression level and prognostic value of PTGES3 and its correlation with potential immunotherapy in LUAD.

**Methods:**

All data were obtained from several databases, including the Cancer Genome Atlas database. Firstly, gene and protein expression of PTGES3 were analyzed using Tumor Immune Estimation Resource (TIMER), R software, Clinical Proteomic Tumor Analysis Consortium (CPTAC), and Human Protein Atlas (HPA). Thereafter, survival analysis was conducted using the R software, Gene Expression Profiling Interactive Analysis 2 (GEPIA2), and Kaplan–Meier Plotter. In addition, gene alteration and mutation analyses were conducted using the cBio Cancer Genomics Portal (cBioPortal) and Catalog of Somatic Mutations in Cancer (COSMIC) databases. The molecular mechanisms associated with PTGES3 were assessed via Search Tool for the Retrieval of Interacting Genes/Proteins (STRING), GeneMANIA, GEPIA2, and R software. Lastly, the role of PTGES3 in immune regulation in LUAD was investigated using TIMER, Tumor-Immune System Interaction Database (TISIDB), and SangerBox.

**Results:**

The gene and protein expression of PTGES3 were elevated in LUAD tissues and compared to the normal tissues, and the high expression of PTGES3 was correlated with cancer stage and tumor grade. Survival analysis revealed that overexpression of PTGES3 was associated with poor prognosis of LUAD patients. Moreover, gene alteration and mutation analysis revealed the occurrence of several types of PTGES3 gene alterations in LUAD. Moreover, co-expression analysis and cross-analysis revealed that three genes, including *CACYBP, HNRNPC*, *and TCP1*, were correlated and interacted with PTGES3. Functional analysis of these genes revealed that PTGES3 was primarily enriched in oocyte meiosis, progesterone-mediated oocyte maturation, and arachidonic acid metabolism pathways. Furthermore, we found that PTGES3 participated in a complex immune regulation network in LUAD.

**Conclusion:**

The current study indicated the crucial role of PTGES3 in LUAD prognosis and immune regulation. Altogether, our results suggested that PTGES3 could serve as a promising therapeutic and prognosis biomarker for the LUAD.

## 1. Introduction

Lung cancer is a leading malignant tumor with a high rate of incidence and mortality worldwide [1]. Lung adenocarcinoma (LUAD), a subtype of non-small cell lung cancer (NSCLC), accounts for approximately 40% of all lung tumors [2]. Traditional therapeutic methods, including surgical excision, radiotherapy, and chemotherapy, have played an important role in the past several decades. However, the 5-year overall survival (OS) rate of LUAD is still <20% [3]. This is possible because the gradual progression of LUAD leads to non-specific symptoms in early-stage patients, so most of the patients are diagnosed at an advanced stage with a poor prognosis. We have recently entered the era of precision medicine [4], and targeted therapy has significant potential in the diagnosis and prognosis of various cancers [5, 6]. Therefore, it is of great significance to identify novel biomarkers of LUAD to improve the treatment and clinical outcomes of LUAD patients.

*PTGES3* gene encodes prostaglandin E synthase 3 enzyme (also known as p23), which acts as a molecular chaperone, localizes to the genomic response elements in a hormone-dependent manner, and disrupts the receptor-mediated transcriptional activation through promoting disassembly of transcriptional regulatory complexes [7]. There has been a recent increase in studies focusing PTGES3 expression in several cancer types. A study reported that PTGES3 is overexpressed in tumor tissues and underexpressed in the adjacent mucosa, in colorectal cancer [8]. Moreover, bioinformatics analysis indicated that PTGES3 played an important role in the progression of osteosarcoma [9]. Another study demonstrated that mRNA expression of PTGES3 is higher in NSCLCs than in normal lung tissues [10]. However, studies on the molecular mechanism and clinical outcome of PTGES3 in NSCLC are limited. In recent years, combination therapy, consisting of immunotherapy, traditional surgery, and radiochemotherapy, has shown great potential in cancer treatment. Immunotherapy has been used for over 100 years including toxins and tumor necrosis factor, vaccines, interleukin 2 (IL-2), antibody therapies, checkpoint inhibitors, oncolytic virus therapy, and chimeric antigen receptor (CAR) T-cell therapy [11]. In a clinical trial, a whole-cell immunotherapy regimen with SV-BR-1-GM cells could suppress breast cancer (BRCA) metastasis [12]. In pancreatic cancer (PC), cancer-associated fibroblasts could modify the tumor microenvironment (TME) to facilitate cancer immune escape, which could be a target for immunotherapy [13]. In LUAD, *α*5 nicotinic acetylcholine receptors could mediate STAT3/PD-L1 to regulate cell migration and invasion [14], while high THBS2 expression predicted poor outcomes of immunotherapy response and post-treatment prognosis [15]. Thus far, several studies have been conducted on immunotherapy for LUAD; however, information on the role of PTGES3 in clinical outcomes and immune regulation in LUAD is limited.

In this study, we identified the gene and protein expression levels of PTGES3 in LUAD and normal tissues based on the Cancer Genome Atlas (TCGA) and other public databases. Thereafter, we evaluated the role of PTGES3 on the clinical characteristics and survival of LUAD patients. Moreover, we explored gene alterations and mutations of PTGES3 in LUAD. Furthermore, we conducted co-expression and enrichment analyses of PTGES3. Lastly, we performed immune-related analyses to investigate the role of PTGES3 in immune regulation in LUAD. We believe that the results of our study would provide the basis for the development of immunotherapy for LUAD treatment.

## 2. Materials and Methods

### 2.1. Data Extraction

The transcriptome profile and clinical data of LUAD patients (Table 1), including TNM stage (T1: tumor size ≤3 cm, T2: 3–5 cm, T3: 5–7 cm; T4: >7 cm; N0: no lymph node metastasis, N1: the involved lymph nodes are mainly located around the tumor, N2: the involved lymph nodes have reached the central region, and N3: the involved lymph nodes have reached the contralateral or supraclavicular lymph nodes; M0: no distant metastasis and M1: distant metastasis), pathologic stage (stage I: T1N0M0 and T2N0M0; stage II: T1N1M0, T2N0M0, T2N1M0, and T3N0M0; stage III: T1N2M0, T1N3M0, T2N2M0, T2N3M0, T3N1M0, T3N2M0, T3N3M0, T4N0M0, T4N1M0, T4N2M0, and T4N3M0; stage IV: M1), primary therapy outcome, gender, age, residual tumor, anatomic neoplasm subdivision, and smoking status, were extracted from TCGA database (https://portal.gdc.cancer.gov/). The RNAseq data in FPKM (Fragment Per Kilobase of transcript per Million mapped reads) format were converted to TPM (Transcripts Per Million) format and then log2 transformed for further analysis. The RNA expression data were conducted with mean ± SD by R software (version 3.6.3).

### 2.2. Gene and Protein Expression

The Tumor Immune Estimation Resource (TIMER, https://cistrome.Shinyapps.io/timer/) was used to analyze the differential expression of PTGES3 across various cancers and corresponding normal tissues based on TCGA database. Thereafter, the R software was used to explore PTGES3 expression in LUAD in unpaired and paired samples by the “ggplot2” package. The PTGES3 proteomic expression profile based on sample type, cancer stage, age, gender, weight, tumor grade, and tumor histology in LUAD was obtained from Clinical Proteomic Tumor Analysis Consortium (CPTAC, https://proteomics.cancer.gov/programs/cptac). In addition, the Human Protein Atlas (HPA, https://proteinatlas.org/) was used to explore PTGES3 expression at the translation level under the “Tissue” and “Pathology” module. *P* < 0.05 was regarded as statistically significant.

### 2.3. Survival Analysis

Receiver operating characteristic (ROC) curve was plotted to evaluate the predictive power of PTGES3 by “pROC” and “ggplot2” packages. Thereafter, “survival” and “survminer” packages were used to explore the role of PTGES3 in the OS and disease-specific survival (DSS) in LUAD. In addition, the Gene Expression Profiling Interactive Analysis 2 (GEPIA2, http://gepia2.cancer-pku.cn/) and Kaplan–Meier (KM) Plotter (http://kmplot.com/analysis/) were used to further validate these results. *P* < 0.05 was considered as statistically significant.

### 2.4. Gene Alteration and Mutation Analysis

The cBio Cancer Genomics Portal (cBioPortal, http://cbioportal.org/) and Catalog of Somatic Mutations in Cancer (COSMIC, https://cancer.sanger.ac.uk/cosmic/) were used to analyze the alteration and mutations of *PTGES3* in LUAD.

### 2.5. Gene Network and Enrichment Analysis

We used the Search Tool for the Retrieval of Interacting Genes/Proteins (STRING; http://string-db.org, v11.5; medium confidence: 0.400) database and GeneMANIA (http://genemina.org/) to identify 20 PTGES3-interacting genes. In contrast, the GEPIA2 tool was used to obtain the top 100 PTGES3-correlated genes based on TCGA data. Thereafter, we conducted a cross-analysis between PTGES3-correlated genes and PTGES3-interacting genes via a Venn diagram using Jvenn (http://bioinfo.genotoul.fr/jvenn). The common genes were then examined with TIMER, GEPIA2, and R software and also subjected to functional enrichment analyses, including Kyoto Encyclopedia of Genes and Genomes (KEGG) pathway analysis and Geno Oncology (GO) enrichment analysis, using “ggplot2” and “ClusterProfiler” packages. *P* < 0.05 was regarded as statistically significant.

### 2.6. Immune-Related Analysis

The TIMER was used to assess the correlation between PTGES3-expression/cumulative survival (CS) and immune infiltrates, including B cells, CD8+ T cells, CD4+ T cells, macrophages, neutrophils, and dendritic cells (DCs), in LUAD. In addition, Tumor-Immune System Interaction Database (TISIDB, https://cis.hku.hk/TISIDB) was used to explore the correlation between PTGES3 expression and the abundance of 28 tumor-infiltrating lymphocytes (TILs) as well as immune subtypes. Additionally, PTGES3-targeting drugs from the DrugBank database were also investigated in TISIDB. Furthermore, the immune checkpoint (ICP) genes and ESTIMATE score associated with PTGES3 expression were analyzed by SangerBox (http://sangerbox.com/Tool) based on TCGA and Genotype-Tissue Expression databases. *P* < 0.05 was regarded as statistically significant.

## 3. Results

### 3.1. Gene and Protein Expression of PTGES3 in LUAD

**Figure 1:**
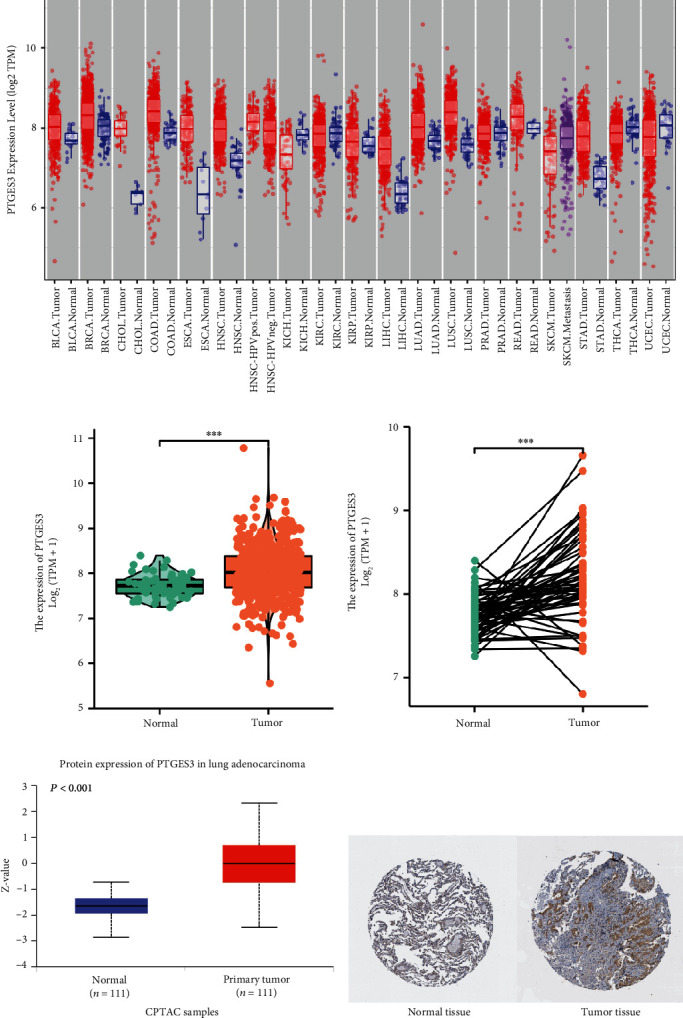
PTGES3 expression levels in human cancers and lung adenocarcinoma. Gene expression levels of PTGES3 in pan-cancer perspective (a). Gene expression levels of PTGES3 in normal tissues and tumor tissues based on unpaired analysis (b) and paired analysis (c). Protein expression levels of PTGES3 in normal tissues and tumor tissues in CPTAC (d) and HPA (e). (∗*P* < 0.05, ∗∗*P* < 0.01, ∗∗∗*P* < 0.001).

The TIMER tool was used to assess the differential expression of PTGES3 in diverse tumor tissues. As seen in Figure 1(a), PTGES3 expression was higher in bladder urothelial carcinoma (BLCA), breast invasive carcinoma (BRCA), cholangiocarcinoma (CHOL), colon adenocarcinoma (COAD), esophageal carcinoma (ESCA), head and neck squamous cell carcinoma (HNSC), liver hepatocellular carcinoma (LIHC), LUAD, lung squamous cell carcinoma (LUSC), rectum adenocarcinoma (READ), and stomach adenocarcinoma (STAD) than those in normal tissues. In contrast, PTGES3 expression was lower in kidney chromophobe (KICH), thyroid carcinoma (THCA), and uterine corpus endometrial carcinoma (UCEC) compared to the normal tissues. For healthy tissues, the expression levels of PTGES3 in bile duct, esophagus, liver, and stomach were significantly lower than those in the other tissues, in which the PTGES3 expression levels are similar. Unpaired (Figure 1(b)) and paired (Figure 1(c)) data analyses revealed that PTGES3 was significantly overexpressed in LUAD than the normal tissues. Furthermore, CPTAC (Figure 1(d)) and HPA (Figure 1(e)) databases revealed that PTGES3 protein expression was upregulated in LUAD compared to the normal tissues.

### 3.2. Association between PTGES3 Expression and Clinical Variables in LUAD

**Figure 2:**
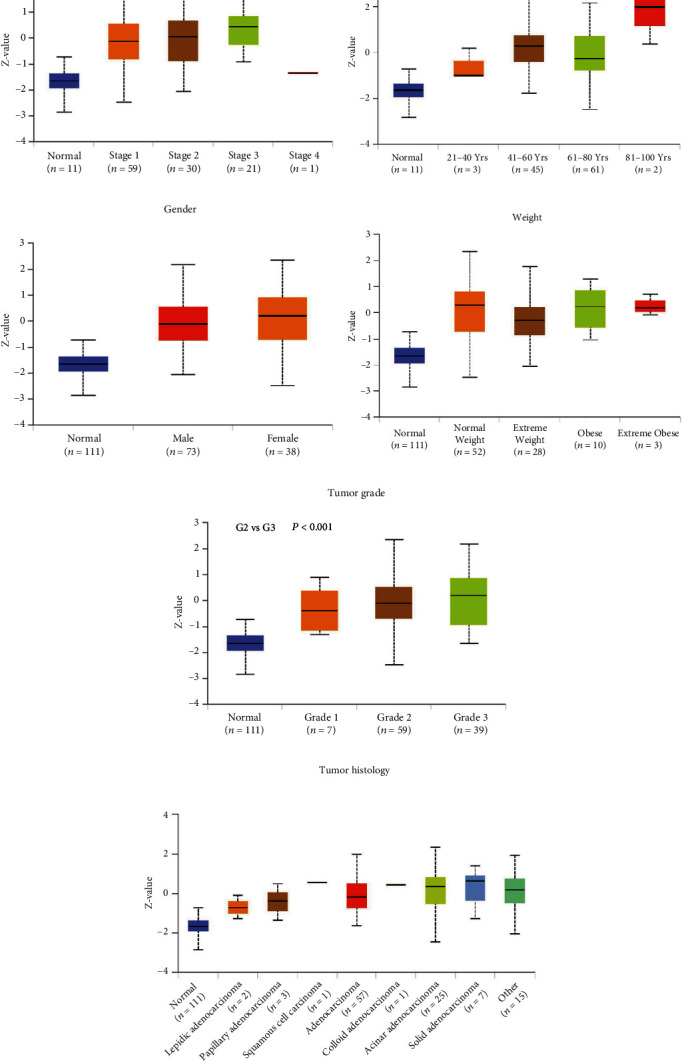
Expression of PTGES3 in different clinical variables including cancer stage (a), age (b), gender (c), weight (d), tumor grade (e), and tumor histology (f).

The CPTAC data further demonstrated that PTGES3 expression level was significantly associated with cancer stage (*P* < 0.05, stage 1 vs. stage 3) and tumor grade (*P* < 0.001, grade 2 vs. grade 3). However, no significant association was found between PTGES3 expression and other clinical characteristics, such as age, gender, weight, and tumor histology (Figure 2).

### 3.3. Survival Analysis

**Figure 3:**
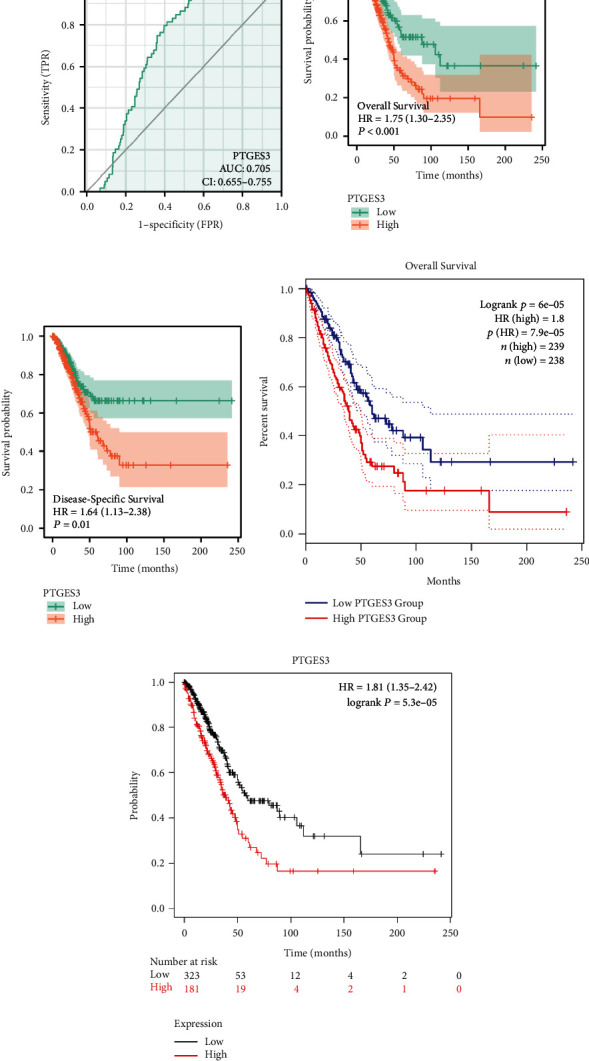
Survival analysis of PTGES3 in LUAD. ROC curve showed promising predictive power (FPR: false positive rate; TPR: true positive rate) (a). High expression of PTGES3 related to poor OS and DSS, respectively, analyzed by R software (b and c). High expression of PTGES3 related to poor OS based on GEPIA2 and Kaplan–Meier Plotter database, respectively (d and e).

TCGA samples were classified into low- and high-expression groups based on the median expression level of PTGES3. ROC curve analysis showed a promising predictive value of PTGES3 expression with an area under curve (AUC) of 0.705 (95% confidence interval, CI: 0.655–0.755; Figure 3(a)). Moreover, our results revealed that high expression of PTGES3 was significantly associated with poor OS (hazard ratio, HR = 1.75, *P* < 0.001; Figure 3(b)) and DSS (HR = 1.64, *P* = 0.01; Figure 3(c)), which were further validated by analyses using GEPIA2 (Figure 3(d)) and KM Plotter (Figure 3(e)).

### 3.4. Gene Alterations and Mutations of PTGES3 in LUAD

**Figure 4:**
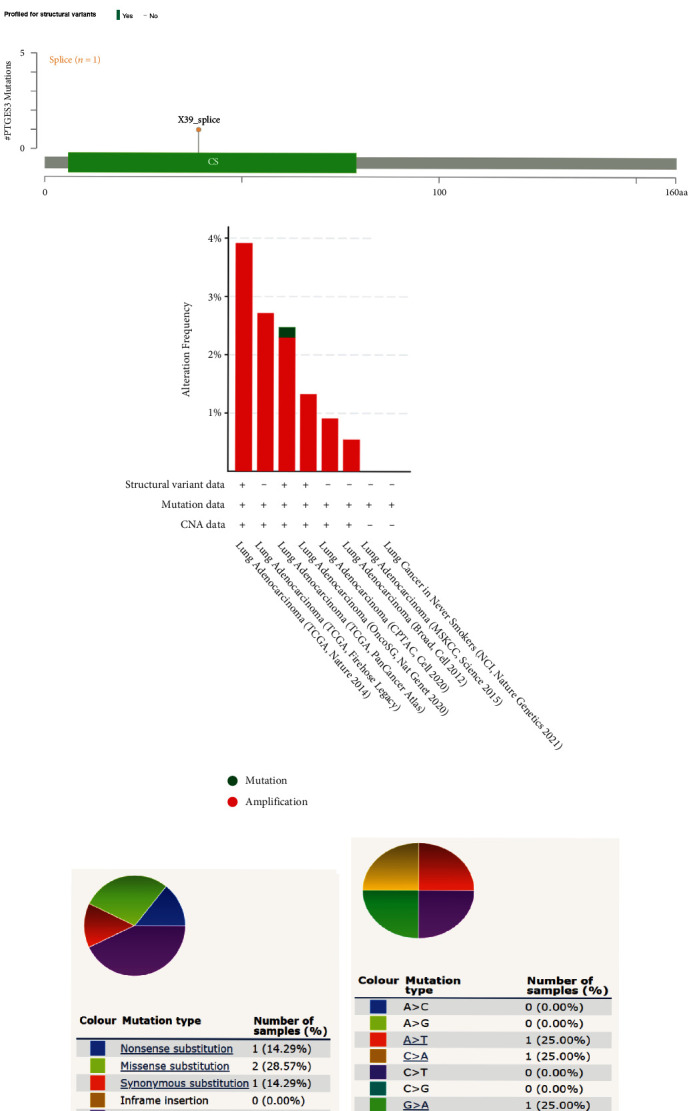
Genomic alterations and mutation of PTGES3 in LUAD based on cBioPortal and COSMIC databases. OncoPrint of PTGES3 expression in LUAD (a). Only splice mutation occurred in PTGES3 (b). Details of PTGES3 gene alteration types in LUAD (c). Overall (d) and substitutions (e) of mutation types in PTGES3.

The cBioPortal database was used to analyze the genomic alterations and mutations of PTGES3 in LUAD, and the results revealed that PTGES3 gene alterations occurred in 2% of LUAD patients (Figure 4(a)) and that only splice mutations occurred in PTGES3 (Figure 4(b)). Moreover, the genomic alteration type in LUAD was primarily amplification (1.93%) rather than mutation (0.046%) (Figure 4(c)). Additionally, analysis by the COSMIC tool revealed the occurrence of three types of mutations in PTGES3 in LUAD, including nonsense (14.29%), missense (28.57%), and synonymous (14.29%) (Figure 4(d)). Among these, the substitutions of A > T, C > A, G > A, and G > T occurred in equal proportions (Figure 4(e)).

### 3.5. Gene Enrichment Analysis

**Figure 5:**
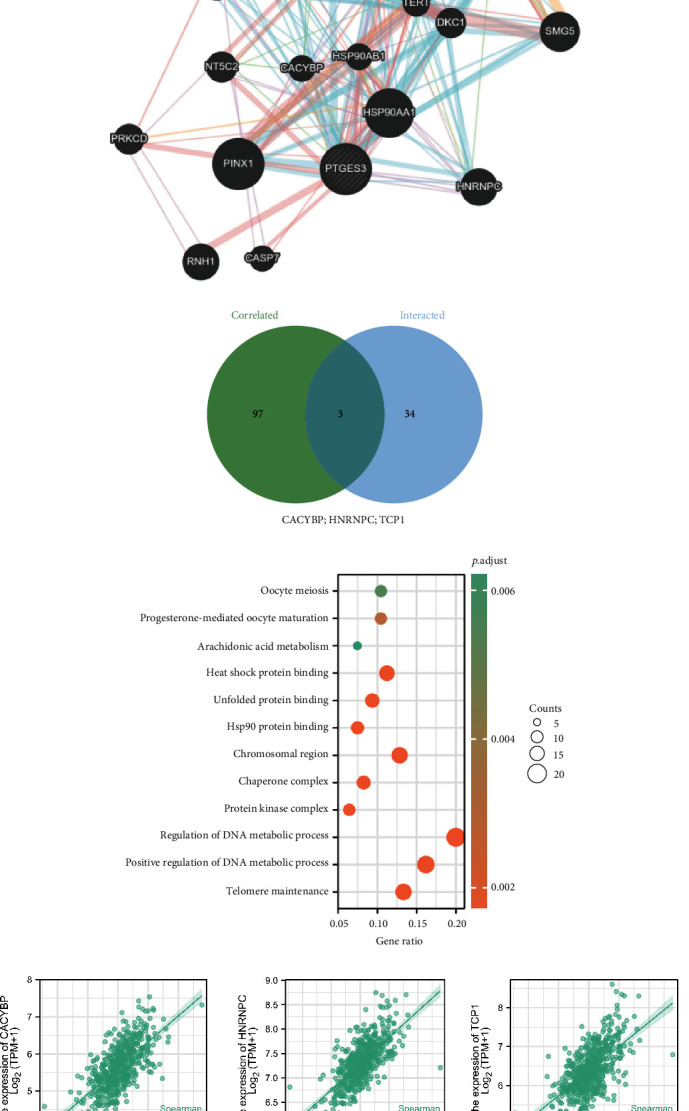
PTGES3-related gene networks and enrichment analysis. Interacted genes of PTGES3 were screened by STRING (a) and GeneMANIA (b) databases. Three common genes including CACYBP, HNRNPC, and TCP1 were identified (c). Enrichment analysis of combined genes (d). PTGES3 was positively correlated with the three common genes based on R software, GEPIA2, and TIMER (e).

We obtained 37 PTGES3-interacted genes from the STRING (Figure 5(a)) and GeneMANIA (Figure 5(b)) and 100 PTGES3-correlated genes from GEPIA2 database (Supplementary 1). Thereafter, we identified three common genes, including *CACYBP*, *HNRNPC*, and *TCP1*, after the cross-analysis of the PTGES3-correlated and PTGES3-interacted genes (Figure 5(c)).  Figure 5(e) illustrates the positive relationships between PTGES3 expression and the expression levels of *CACYBP*, *HNRNPC*, and *TCP1*, as verified by R software, GEPIA2, and TIMER. Furthermore, KEGG enrichment analysis (Figure 5(d)) revealed that the PTGES3-coexpression genes were primarily enriched in oocyte meiosis, progesterone-mediated oocyte maturation, and arachidonic acid (AA) metabolism. While GO enrichment analysis (Figure 5(d)) revealed that PTGES3 was primarily involved in heat shock protein (Hsp) binding, unfolded protein binding, and Hsp90 protein binding in biological processes (BP); chromosomal region, chaperone complex, and protein kinase complex in cellular components (CC); and regulation of DNA metabolic process, positive regulation of DNA metabolic process, and telomere maintenance in molecular functions (MF).

### 3.6. Association between PTGES3 Expression and Immune Regulation in LUAD

**Figure 6:**
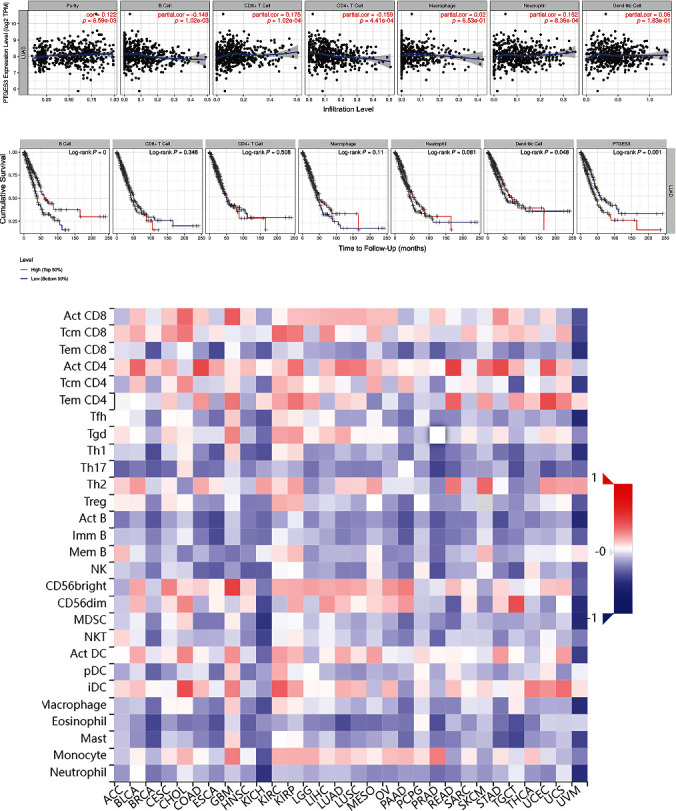
Correlation between PTGES3 expression and immune cell infiltration. PTGES3 expression was significantly related to B cell, CD8+ T cell, CD4+ T cell, and neutrophil (a). The more abundance of B cell was related to favorable cumulative survival, while the more abundance of dendritic cell was related to poor cumulative survival (b). Correlations between expression of PTGES3 and 28 TILs types in pan-cancer perspective based on TISIDB database (c).

**Figure 7:**
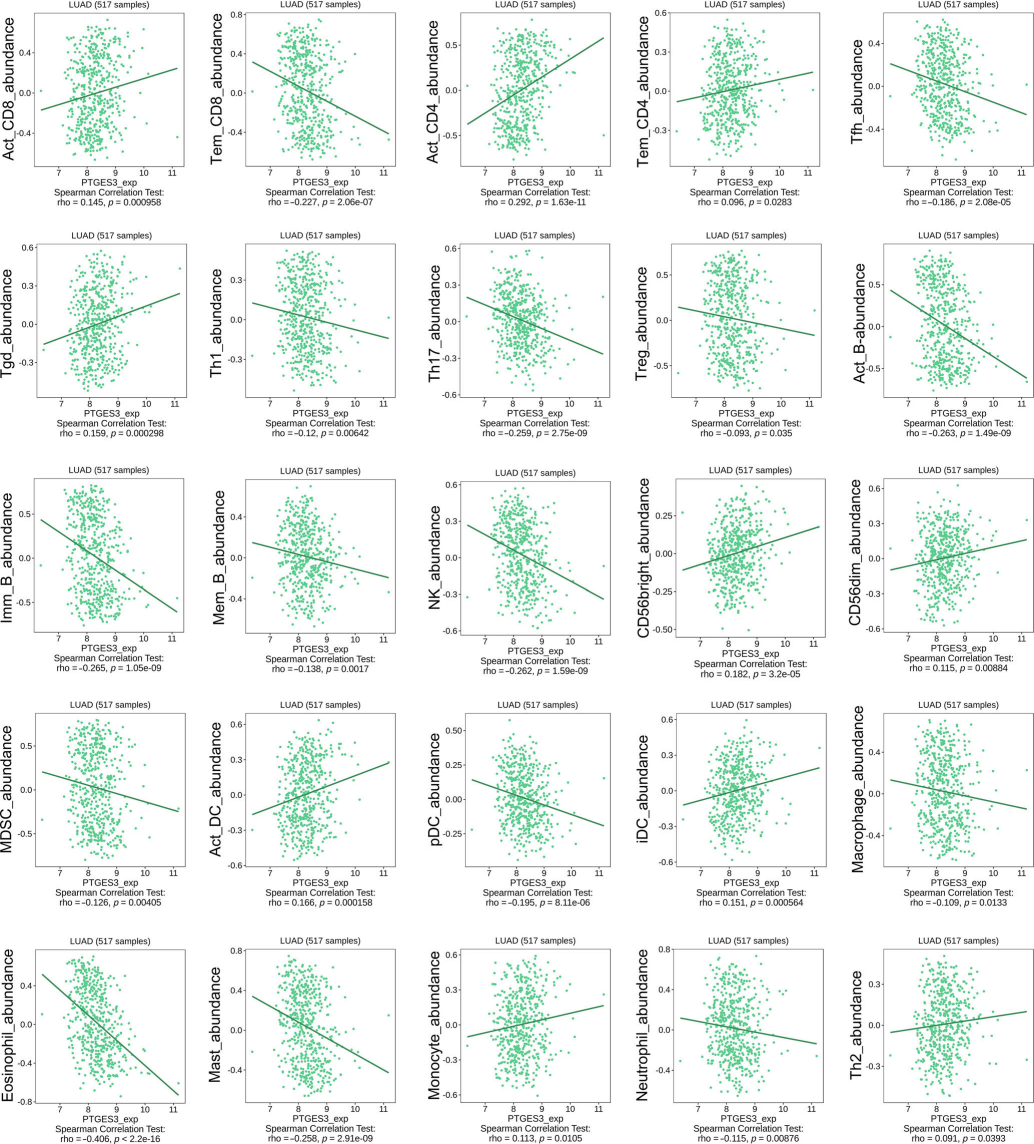
PTGES3 was significantly correlated with Act CD8, Act CD4, Tem CD4, Tgd, CD56bright, CD56dim, Act DC, iDC, monocyte, Th2, Tem CD8, Tfh, Th1, Th17, Treg, Act B, Imm B, Mem B, NK, MDSC, pDC, macrophage, eosinophil, Mast, and neutrophil.

**Figure 8:**
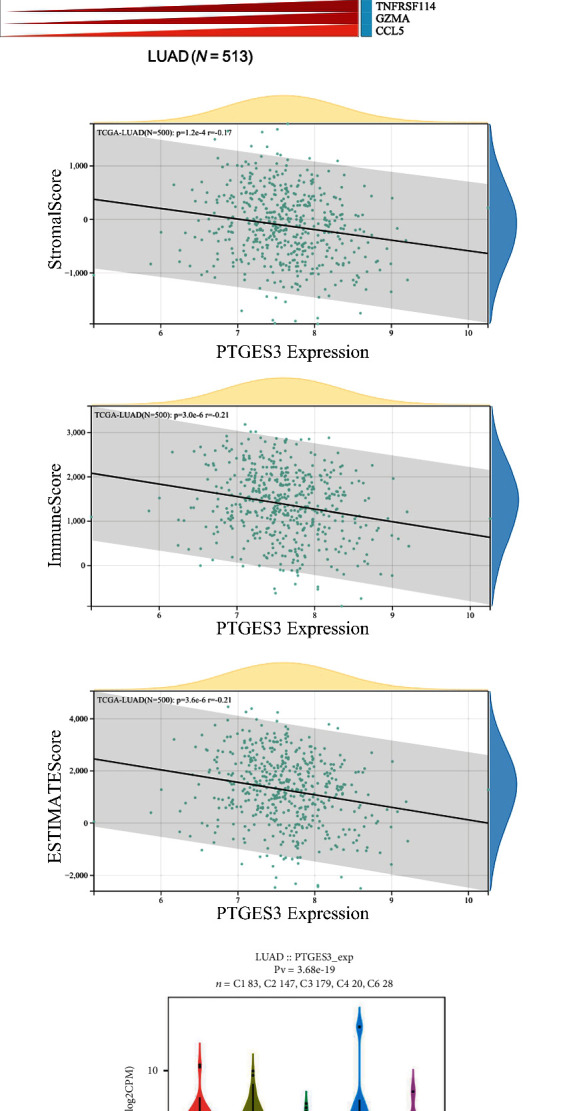
Immune regulation of PTGES3 in LUAD. There were 26 of 60 ICP genes that significantly related to PTGES3 expression in LUAD (a). PTGES3 expression was negatively correlated to stromal score (*P* = 1.2 × 10^−4^), immune score (*P* = 3.0 × 10^−6^), and ESTIMATE score (*P* = 3.6 × 10^−6^) (b). The relationship between PTGES3 expression and immune subtypes (c). Two drugs named Grn 163I and copper targeting to PTGES3 were identified (d).

To understand the role of PTGES3 in immune regulation in LUAD, we analyzed the correlation between PTGES3 expression and six immune cells using the TIMER database. As seen in Figure 6(a), PTGES3 expression was significantly associated with B cell (*r* = −0.149, *P* = 6.59 × 10^−3^), CD8+ T cell (*r* = 0.175, *P* = 1.02 × 10^−4^), CD4+ T cell (*r* = −0.159, *P* = 4.41 × 10^−4^), and neutrophil (*r* = 0.152, *P* = 8.36 × 10^−4^) levels. Moreover, we found that a higher abundance of B cells was associated with favorable CS, while a higher abundance of DCs was associated with poor CS (Figure 6(b)). Additionally, analysis of the correlation between PTGES3 expression and 28 TILs (Figure 6(c)) using the TISIDB database revealed that 25 TILs were significantly associated with PTGES3 in LUAD. Among these, 10 TILs, including activated CD8 T cell (Act CD8), activated CD4 T cell (Act CD4), effector memory CD4 T cell (Tem CD4), gamma delta T cell (Tgd), CD56bright natural killer cell (CD56bright), CD56dim natural killer cell (CD56dim), activated dendritic cell (Act DC), immature dendritic cell (iDC), and monocyte and type 2 helper cell (Th2), showed a positive correlation with PTGES3 expression. In contrast, 15 TILs, including effector memory CD8 T cell (Tem CD8), T follicular helper cell (Tfh), type 1 T helper cell (Th1), type 17 T helper cell (Th17), regulatory T cell (Treg), activated B cell (Act B), immature B cell (Imm B), memory B cell (Mem B), natural killer cell (NK), myeloid-derived suppressor cell (MDSC), plasmacytoid dendritic cell (pDC), macrophage, eosinophil, mast cell (Mast), and neutrophil, showed a negative correlation with PTGES3 expression (Figure 7). Furthermore, among the 60 ICP genes, 28 (13 inhibitor and 15 stimulator genes) were significantly correlated with PTGES3 expression in LUAD (Figure 8(a)). As seen in Figure 8(b), PTGES3 expression was negatively correlated with stromal (*P* = 1.2 × 10^−4^), immune (*P* = 3.0 × 10^−6^), and ESTIMATE (*P* = 3.6 × 10^−6^) scores in LUAD. In addition, the immune subtypes in LUAD were divided into: C1 (wound healing), C2 (Interferon-*γ* dominant), C3 (inflammatory), C4 (lymphocyte depleted), C5 (immunologically quiet), and C6 (Transforming Growth Factor-*β* dominant) (Figure 8(c)). Lastly, we found two drugs that targeted PTGES3, including Grn 163l and copper (Figure 8(d)).

## 4. Discussion

In the present study, we found that the mRNA and protein expression of PTGES3 were higher in LUAD tissues compared to be normal tissues. Additionally, we found the overexpression of PTGES3 was positively associated with cancer stage and tumor grade in LUAD. In addition, the ROC curve suggested that high expression of PTGES3 was associated with poor OS and DSS in LUAD and that PTGES3 could serve as a promising predictive biomarker for survival analysis. Moreover, co-expression and enrichment analyses revealed that PTGES3 was involved in a complex regulatory network in LUAD. Furthermore, we discovered that PTGES3 played an important role in immune regulation in LUAD. Altogether, our results suggest that PTGES3 could serve as a potential prognostic biomarker for immunotherapy in LUAD.

*PTGES3* encodes prostaglandin E synthase enzyme, and the deregulation of these enzymes in the prostaglandin-endoperoxide synthase pathway by inhibition of COX-2 activity leads to an abnormal level of pro- and anti-inflammatory signals associated with tumorigenesis. Prostaglandin, produced in a COX2-dependent manner, could act on the epithelium to regulate intravasation and immune cell function in malignant cells [16, 17]. Previous studies have revealed that PTGES3 participates in the regulation of various diseases, including pediatric recurrent abdominal pain, oscillatory shear stress, dyspepsia, and cancers [18–20]. In recent years, there has been an increasing interest in the tumorigenic role of PTGES3 in multiple cancer types, including colorectal cancer, prostate cancer, and acute lymphoblastic leukemia [8, 22]. For instance, PTGES3 was reported to regulate the function of oncoprotein telomerase, which could affect epithelial cell transformation and human mammary epithelial cell immortalization. In prostate cancer, PTGES3 was reported to induce the androgen receptor activity and chromatin binding to promote tumorigenesis [22]. Interestingly, several *in vitro* and bioinformatics studies revealed that PTGES3 is upregulated in NSCLC [10, 23]. However, the expression level and prognostic value of PTGES3 in LUAD are still unknown. Consistent with the results of previous studies, our study revealed that PTGES3 was abnormally expressed in many cancers. Additionally, it revealed that mRNA and protein expression of PTGES3 were significantly upregulated in LUAD and that its high expression was associated with cancer stage and tumor grade. Similar results were observed in a previous study, which revealed that high PTGES3 expression was associated with the stage of endometrioid endometrial cancer [24]. Interestingly, we found that there was no significant difference on the expression level of PTGES3 between tumor and normal tissues in several cancer types, including kidney renal clear cell carcinoma (KIRC), kidney renal papillary cell carcinoma (KIRP), and prostate adenocarcinoma (PRAD), which belongs to genitourinary cancer. The present studies were still superficial. The reason that why these types of tumors do not need PTGES3 to regulate and the relationship between PTGES3 and urinary system have not been clearly described. Therefore, in-depth studies were needed. Furthermore, the prognostic value of PTGES3 in LUAD was evaluated through TCGA, GEPIA2, and KM Plotter databases, which demonstrated that the upregulation of PTGES3 in cancer tissues was correlated with poor OS and DSS. Bioinformatics analysis of COX/prostaglandin pathway genes in BRCA demonstrated that PTGES3 had a significant negative effect on survival [25]. In addition, PTGES3 was found to be a hub gene enriched in cell cycle-related pathways in cholangiocarcinoma (CCA), which could serve as a prognostic marker [26]. Therefore, we speculated that PTGES3 could serve as a prognostic predictor for LUAD.

To further understand the molecular mechanisms of PTGES3 in LUAD, we used STRING, GeneMANIA, and GEPIA2 to identify the PTGES3-interacted and coexpression genes and used R software to conduct enrichment analyses. We found three common genes, including *CACYBP*, *HNRNPC,* and *TCP1* through the cross-analysis between the PTGES3-correlated and -interacted genes. Although correlations between PTGES3 and these three genes have not been reported, several studies imply an intrinsic connection between these genes. A study reported an interaction between CACYBP/SIP and Hsp90, suggesting that CACYBP/SIP participated in the regulation of Hsp90 chaperone activity, in which PTGES3 is a critical candidate [27, 28]. Furthermore, in acute lung injury, CACYBP could modulate cell signaling *in vivo* [29]. Moreover, CACYBP was upregulated in NSCLC cells than the human bronchial epithelial cells, further promoting cell proliferation and invasion via the AKT signal pathway [30]. HNRNPC, a member of the HNRNP family, serves as a RNA-binding protein in N6-methyladenosine (m6A) methylation, which is involved in the occurrence and progression of multiple cancers. In prostate cancer, HNRNPC could promote proliferation and metastasis and inhibit prognosis [31]. Additionally, upregulation of HNRNPC in metastatic *in vivo* models could accelerate tumorigenesis in PC [32]. Moreover, bioinformatics analysis and immunohistochemical staining revealed that HNRNPC was negatively associated with the OS in LUAD [33]. Therefore, we speculate that PTGES3 and HNRNPC could synergistically promote cancer occurrence and development, which needs to be explored further. TCP1 is an oncogene in various cancers, which improves cell proliferation through the activation of the PI3K/AKT/mTOR signaling pathway [34]. In hepatocellular carcinoma, TCP1 regulates cell proliferation and migration by modulating the Wnt7b/*β*-catenin pathway [35]. However, the role of TCP1 in LUAD and its association with PTGES3 are not fully understood. Enrichment analysis revealed that the PTGES3-coexpression genes were associated with AA metabolism pathway. In oral squamous cell carcinoma, the AA metabolism pathway contributed to lipid oxidation, inflammation, proliferation, and migration, which were associated with tumorigenesis [36]. Moreover, targeting enzymes related to AA metabolism and cancer inflammation, including cPLA(2), COXs, and LOXs, for cancer radiotherapy, improved prognosis [37]. A previous study reported that prostanoid signaling is a part of the AA metabolism pathway [38]. However, the role of PTGES3 in the regulation of AA metabolism in LUAD is unclear. Functional analysis, in our study, demonstrated that PTGES3 participated in complex network in LUAD, with several genes and pathways.

In recent years, many studies have focused on immunotherapy for cancer treatment. The immunobiology of TME is associated with several modulators, including TILs, vasculature, tumor location, and tumor stroma [39–41]. A few studies have reported the promising role of PTGES3 in cancer immunity. A previous study demonstrated that the RNA-binding function of PTGES3 could be beneficial for the regulation of macrophage phagocytotic activity and migration [42]. In BRCA, upregulation of PTGES3 was significantly correlated to CD8+ T cell abundance in TME, suggesting that PTGES3 could be an immune regulator. In addition, it was revealed that PTGES3 was negatively associated with immunoinhibitors, immunostimulators, and Major Histocompatibility Complex molecules in cervical cancer [43]. However, the relationship between PTGES3 expression and immune regulation in LUAD has not yet been studied. In the current study, we conducted immune-related analysis, including immune infiltrates, ICPs, immune subtypes, ESTIMATE scores, and PTGES3-targeting drugs, based on several public databases. We found that PTGES3 was negatively associated with B cell and CD4+ T cell abundance and positively associated with CD8+ T cell and neutrophil abundance in LUAD. Interestingly, the low abundance of B cells was associated with poor CS. Moreover, increasing evidence has demonstrated that low levels of infiltrating B cells are correlated with poor outcomes in HNSC [44], gastric cancer [45], hepatocellular carcinoma [46], and BRCA [47]. In contrast, high levels of infiltrating B cells are correlated with shorter survival in renal cell cancer [48]. Thus, it was speculated that PTGES3 affected the prognosis of LUAD patients by regulating the immune infiltrating cells such as B cells. Furthermore, we also investigated the correlation between PTGES3 and ICP genes and found that PTGES3 was significantly associated with approximately half of the ICP inhibitors and stimulators, including CD274 and CD276. A previous study revealed that CD274 and CD276 showed potential effectiveness in immunotherapy for LUAD patients with a history of smoking [49]. In addition, we found that PTGES3 was negatively correlated with stromal, immune, and ESTIMATE scores and found that upregulation of PTGES3 was correlated with low infiltration of immune and stromal cells but high tumor purity in LUAD. Furthermore, it was reported that PTGES3 affected sensitivity of chemotherapeutic drugs. PTGES3 has been reported to reduce geldanamycin sensitivity in mammalian cancer cells [22]. In the present study, we identified two PTGES3-targeting drugs, including Grn 163I and copper. Copper is a transition metal in the human body and plays an important role in many enzymes, including cytochrome c oxidase, monoamine oxidase, and superoxide dismutase. Grn 163l is a novel anti-cancer drug that has been reported to inhibit telomerase in lung cancer and BRCA [50, 51]. These results suggest that PTGES3 can serve as a therapeutic target for immunotherapy in LUAD.

Although our findings provide novel insight into the correlation between PTGES3 and LUAD, there were several limitations to our study. Firstly, *in vitro* and *in vivo* studies should be performed to elucidate the MF of PTGES3 in LUAD and to further validate our results. Secondly, more clinical factors should be considered to promote the clinical application of PTGES3-targeting treatments for LUAD.

## 5. Conclusion

In this study, we found that PTGES3 expression is upregulated in LUAD and that its overexpression is closely correlated with short survival in LUAD patients. Moreover, we found that PTGES3 played an important role in the immune regulation network in LUAD, suggesting that PTGES3 can serve as a promising therapeutic and prognostic target for immunotherapy in LUAD.

## Figures and Tables

**Table 1:** Clinical characteristics of lung adenocarcinoma (TCGA).

Characteristic	Low expression of PTGES3	High expression of PTGES3	*p*
*n*	267	268	
T stage, *n* (%)			
T1	104 (19.5%)	71 (13.3%)	0.009
T2	128 (24.1%)	161 (30.3%)	
T3	25 (4.9%)	23 (4.3%)	
T4	7 (1.3%)	12 (2.3%)	
N stage, *n* (%)			
N0	184 (35.5%)	164 (31.6%)	0.069
N1	38 (7.3%)	57 (11%)	
N2	32 (6.2%)	42 (8.1%)	
N3	1 (0.2%)	1 (0.2%)	
M stage, *n* (%)			
M0	165 (42.7%)	196 (50.8%)	0.057
M1	6 (1.6%)	19 (4.9%)	
Pathologic stage, *n* (%)			
Stage I	159 (30.2%)	135 (25.6%)	0.029
Stage II	55 (10.4%)	68 (12.9%)	
Stage III	40 (7.6%)	44 (8.3%)	
Stage IV	7 (1.3%)	19 (3.6%)	
Primary therapy outcome, *n* (%)			
PD	28 (6.3%)	43 (9.6%)	0.070
SD	21 (4.7%)	16 (3.6%)	
PR	3 (0.7%)	3 (0.7%)	
CR	187 (41.9%)	145 (32.5%)	
Gender, *n* (%)			
Female	157 (29.3%)	129 (24.1%)	0.017
Male	110 (20.6%)	139 (26%)	
Age, *n* (%)			
≤65	133 (25.8%)	122 (23.6%)	0.427
>65	126 (24.4%)	135 (26.2%)	
Residual tumor, *n* (%)			
R0	176 (47.3%)	179 (48.1%)	0.019
R1	3 (0.8%)	10 (2.7%)	
R2	0 (0%)	4 (1.1%)	
Anatomic neoplasm subdivision, *n* (%)			
Left	105 (20.2%)	100 (19.2%)	0.667
Right	154 (29.6%)	161 (31%)	
Anatomic neoplasm subdivision 2, *n* (%)			
Central lung	32 (16.9%)	30 (15.9%)	0.474
Peripheral lung	57 (30.2%)	70 (37%)	
Smoker, *n* (%)			
No	35 (6.9%)	39 (7.5%)	0.762
Yes	228 (43.4%)	220 (42.2%)	
Age, median (IQR)	65 (58.5, 72)	56 (59, 72)	0.449

## Data Availability

The data used to support the findings in this study are available on the public online websites mentioned in this paper.

## Supplementary Materials

Supplementary Materials
*Supplementary 1*: The top 100 PTGES3-correlated genes identified using Gene Expression Profiling Interactive Analysis 2 database.
